# Adaptive Robotic Control Driven by a Versatile Spiking Cerebellar Network

**DOI:** 10.1371/journal.pone.0112265

**Published:** 2014-11-12

**Authors:** Claudia Casellato, Alberto Antonietti, Jesus A. Garrido, Richard R. Carrillo, Niceto R. Luque, Eduardo Ros, Alessandra Pedrocchi, Egidio D'Angelo

**Affiliations:** 1 NeuroEngineering and Medical Robotics Laboratory, Department of Electronics, Information and Bioengineering, Politecnico di Milano, Milano, Italy; 2 Brain Connectivity Center, Istituto di Ricovero e Cura a Carattere Scientifico Istituto Neurologico Nazionale Casimiro Mondino, Pavia, Italy; 3 Department of Brain and Behavioral Sciences, University of Pavia, Pavia, Italy; 4 Department of Computer Architecture and Technology, Escuela Técnica Superior de Ingegnerías Informática y de Telecomunicación, University of Granada, Granada, Spain; University of Genova, Italy

## Abstract

The cerebellum is involved in a large number of different neural processes, especially in associative learning and in fine motor control. To develop a comprehensive theory of sensorimotor learning and control, it is crucial to determine the neural basis of coding and plasticity embedded into the cerebellar neural circuit and how they are translated into behavioral outcomes in learning paradigms. Learning has to be inferred from the interaction of an embodied system with its real environment, and the same cerebellar principles derived from cell physiology have to be able to drive a variety of tasks of different nature, calling for complex timing and movement patterns. We have coupled a realistic cerebellar spiking neural network (SNN) with a real robot and challenged it in multiple diverse sensorimotor tasks. Encoding and decoding strategies based on neuronal firing rates were applied. Adaptive motor control protocols with acquisition and extinction phases have been designed and tested, including an associative Pavlovian task (Eye blinking classical conditioning), a vestibulo-ocular task and a perturbed arm reaching task operating in closed-loop. The SNN processed in real-time mossy fiber inputs as arbitrary contextual signals, irrespective of whether they conveyed a tone, a vestibular stimulus or the position of a limb. A bidirectional long-term plasticity rule implemented at parallel fibers-Purkinje cell synapses modulated the output activity in the deep cerebellar nuclei. In all tasks, the neurorobot learned to adjust timing and gain of the motor responses by tuning its output discharge. It succeeded in reproducing how human biological systems acquire, extinguish and express knowledge of a noisy and changing world. By varying stimuli and perturbations patterns, real-time control robustness and generalizability were validated. The implicit spiking dynamics of the cerebellar model fulfill timing, prediction and learning functions.

## Introduction

The cerebellum plays an essential role in adaptive motor control including fast and smooth coordination, on-line adaptation of movement, and classical conditioning of various behavioral responses [1]. The cerebellum is thought to assist these operations by predicting system future states through associative learning of precise timing between subsequent components of the sensorimotor process [2], [3]. To develop a comprehensive theory of sensorimotor learning and control, it is crucial to determine the neural basis of coding and plasticity embedded into the cerebellar neural circuit [4].

As a first step in assessing the role of cerebellar plasticity in learning, simplified analog models were developed and tested in the context of various sensorimotor tasks. These models were based on the Adaptive Filter Model derived from the Marr-Albus Motor Learning Theory [5], [6], [7], [8], [9], [10]. In these models, parameter adaptation was driven by the correlation of a sensory error signal with a motor command signal, which were proposed to correspond to climbing fiber and mossy fiber signals, respectively. Learning was proposed to occur as long-term synaptic plasticity at the parallel fiber - Purkinje cell synapses in the form of long-term depression under instructive control by climbing fibers. Simulations carried out with these models suggested that a control scheme incorporating cerebellum adaptation played indeed a critical role for point-to-point arm movement and for prism glasses compensation in throwing at a target [11], [12], for anticipatory grip force modulation [13], and for Pavlovian collision avoidance [14]. While computational simulations guarantee repeatability over trials and therefore a systematic evaluation of the control scheme, testing with real robots is needed to assess the robustness and generalizability of models in closed-loop conditions, in which unwanted perturbations disturb learning and control. To this purpose, analog adaptive models of cerebellum were tested as controllers of real robots performing collision avoidance and eye movement tasks [15], [16], [17], [18]. These robotic models were able to learn smooth motor responses and thus confirmed that the cerebellum could operate as a predictive controller under closed-loop conditions.

Although analog models provided insights into the overall cerebellar process of learning and control, the cerebellar network operates on the basis of implicit computations with spikes. A bio-realistic approach to sensorimotor learning and control requires therefore to develop adaptive Spiking Neural Networks (SNNs). SNNs can more directly cross-validate computational principles derived from cell physiology, as they can incorporate the timing based on neuronal firing [19]. Since biological organisms set up effective control systems using ensembles of interconnected firing neurons, SNNs clearly do have the potential to produce effective control systems for robots [20]. Generic SNNs were applied to control sensorimotor tasks in simulations ranging from eye [21] to multi-joint arm movements [22]. A cerebellar SNN was used to demonstrate time-control in delay-eyeblink conditioning [23] and simultaneous time- and gain-control in optokinetic response adaptation [24] in computational simulations.

A critical issue is that learning has to be inferred from the interaction of an embodied system with its environment, which implies controlling a robot with SNNs in real-time. Generic SNN-based controllers were used to drive mobile robots [25], [26], [27] with training methods using genetic algorithms. Cerebellar-based SNN controllers were developed and tested in real robots performing Pavlovian conditioning tasks [28], [29]. In particular, a large-scale cerebellar SNN based on the liquid-state machine model and expressing a single plasticity site at the parallel fiber - Purkinje cell synapses [29] was able to actuate a robot to hit a ball with proper timing, i.e. shortly in advance of ball arrival. It was not clear, however, whether the SNN controller was able to elaborate general control functions using realistic spiking representations of neurons and performing versatile smooth adaptive timing and gain control. Indeed, very often the models using cerebellar principles, more or less detailed and realistic, have been designed specifically for one single task [30], [31], [32], [33], [34], while the real cerebellum is good at learning a wide variety of tasks, going from stimuli associations to adaptive sensorimotor transformations and coordination.

In this framework, we have coupled a realistic cerebellar SNN with a real robot and challenged the system in multiple sensorimotor tasks. The cerebellar SNN has been derived from a detailed cerebellar network [35], designed to run in real-time through acceleration technologies [36], and interfaced with a robotic controller using Analog/Digital and Digital/Analog transformation algorithms. The neurorobot has been tested in multiple learning paradigms including Pavlovian associative tasks and continuous closed-loop tasks (vestibulo-ocular reflex and upper limb reaching under perturbing force-fields). The neurorobot adapted its performance and efficiently controlled the different tasks exploiting the internal firing dynamics of the neural network and successfully dealt with changing environmental conditions and with hard real-time constraints. Thus, the cerebellar SNN operated as a generalized predictive controller, showing that the SNN-robot provides a flexible and modular platform to link low-level brain circuit operations with high-level behavioral functions.

## Methods

### Spiking cerebellar model

**Figure 1:**
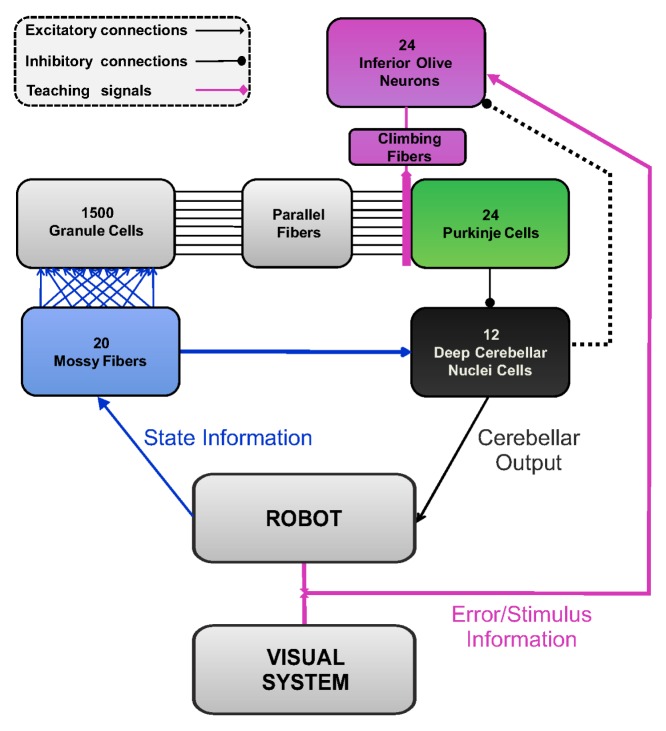
Cerebellar SNN. The computational model applied for creating the cerebellar spiking neural network embedded into the controller of the robotic platform.

The SNN was built exploiting the Event-Driven simulator based on Look-Up Table (EDLUT) [37], which is an open-source computer application for simulating SNNs. The EDLUT neural simulator is open source and available at https://code.google.com/p/edlut/. The three experimental set ups built in a simulator environment are also made available in ModelDB http://senselab.med.yale.edu/modeldb/enterCode.asp?model=167414. EDLUT operates by compiling the dynamic responses of pre-defined cell models into lookup tables, thus allowing real-time performance. The present cerebellar SNN architecture represents a cerebellar modular element and consisted of (Fig. 1):

- 20 Mossy Fibers (MFs) as input.
- 1500 Granule cells (GRs); as in real cerebellum, each GR receives four excitatory input connections selected randomly from MFs with constant synaptic weights. When an input signal arrives, a spatiotemporal activity pattern is generated and the population of active neurons in the granule layer changes in time according to the received MF signals. The output of these GRs are conveyed through the Parallel Fibers (PFs).
- 24 Inferior Olive cells (IOs); each IO sends a Climbing Fiber (CF) to one Purkinje cell (PC).
- 24 Purkinje Cells (PCs); each GR is connected to 80% of the PCs, through the PFs.
- 12 Deep Cerebellar Nuclei cells (DCNs); each DCN receives inhibitory connections from 2 PCs and excitatory connections from all the 20 MFs.

In the SNN, synaptic adaptation occurs at PF–PC connections as a change in synaptic conductance (w) through a spike-timing dependent plasticity rule inducing either Long-Term Depression (LTD) or Long-Term Potentiation (LTP) [38]. LTD results from coincident PF- and CF-activation, taking into account all the PF spikes falling within a given time window preceding the CF spikes (*eligibility trace*). This is obtained convolving the PF activity with a kernel function taking into account the physiological delay of the neural circuit, which ranges from 50 to 150 ms [39]. Conversely, LTP results from PF-stimulation alone [36]. The learning rule is:
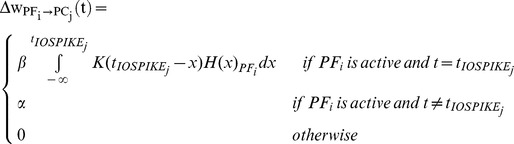
where



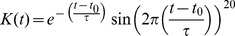


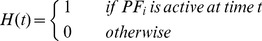
where β is the LTD constant (<0), α is the LTP constant (>0); *t_IOSPIKEj_* is the time when the corresponding CF_j_ emits a spike; *K* is the integral kernel function; *τ* is used in order to normalize the arguments in the learning rule and it is set to 1 second; *t_0_* corresponds to the physiological delay of the neural circuit and it is set to 100 ms, as dictated by biology.

α and β can be tuned, with α lower than the absolute value of β, otherwise LTP, constantly generated when a state-related activity comes from GRs, could counterbalance and nullify LTD effects. We have explored different α and β values within reasonable ranges, evaluating their effect on the acquisition effectiveness and rate, on the late acquisition stability and on the extinction effectiveness and rate, in computational simulations of eye blinking conditioning (see below *Protocols* section).

In the continuous closed-loop tasks, topography is maintained at level of IOs, PCs and DCNs: a group of IOs carries information about the error (positive and negative separately) of a specific Degree of Freedom (DoF) and projects on a corresponding group of PCs. These PCs then project to a group of DCNs, which produce an agonist or antagonist motor action (positive or negative torque) on that specific DoF.

### Set-up

The main robot was a Phantom Premium 1.0 (SensAble™), with 3 DoFs, each equipped with digital encoders and controllable by torque commands. This robot was integrated with an optical tracking system (VICRA-Polaris, NDI™) acquiring marker-tools at 20 Hz. A secondary robotic device (Phantom Omni, SensAble™) was synchronized into the controller to add desired perturbations in the different tested paradigms. The controller, developed ad-hoc in C++, exploited the low-level access provided by the Haptic Device Application Programming Interface and sent the torque signals to the joints by servo loops (HDCALLBACKS) executed in high-priority threads at 1 kHz. For the tracking device, the low-level libraries from Image-Guided Surgery Toolkit (http://www.igstk.org/), based on Request-Observer patterns, were used to acquire the visual information. The cerebellar model was embedded into the C++ controller and its updating ran at 1 kHz. All the experiments were performed on a desktop PC (Intel Core i7-2600 CPU @3.40 GHz).

### Protocols

**Figure 2:**
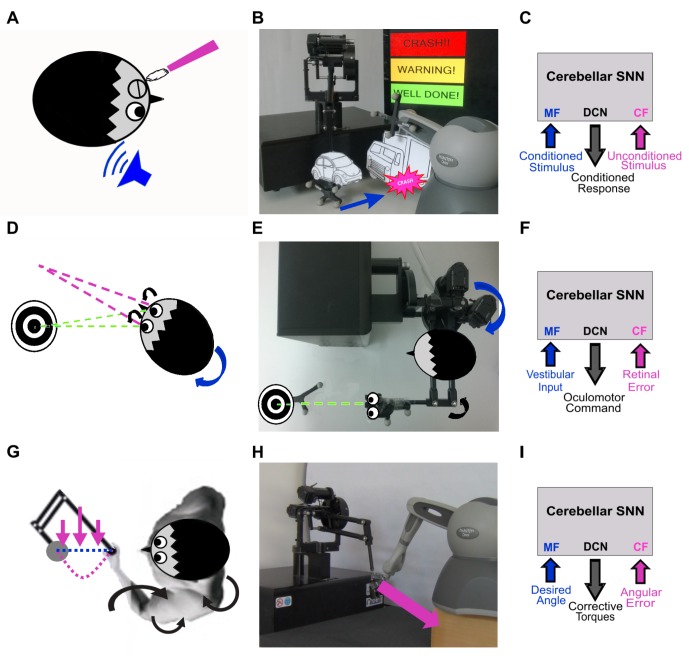
Real robot experiments: neurophysiology, robotic set-up and cerebellar controller. EBCC-like, VOR and upper limb perturbed reaching: on the left column the typical set-up used in neurophysiological studies; in the middle the corresponding set-up used in our robotic tasks and on the right column the cerebellar network with the task-specific input and output signals. (A), (B) and (C): the EBCC-like Pavlovian task is reproduced into the robotic platform as a collision-avoidance task. The CS onset is based on the distance between the moving robot end-effector and the fixed obstacle placed along the trajectory, detected by the optical tracker. The US is the collision event. US is fed into the CF pathway, CS into the MF pathway; the DCNs trigger the conditioned response (anticipated stop). (D), (E) and (F): the VOR is reproduced into the robotic platform by using the second joint of the robotic arm as the head (imposed rotation) and the third joint (determining the orientation of the second link, on which the green laser is placed) as the eye. The disalignment between the gaze direction (i.e. second link orientation) and the environmental target to be looked at (hold and eventually moved by another robotic device) is computed through geometric equations from the optical tracker recording. The image slip is fed into the CF pathway, the vestibular stimulus about the head state into the MF pathway; the DCNs modulate the eye compensatory motion. (G), (H) and (I): the perturbed reaching is reproduced into the robotic platform by applying a viscous force field on the moving robotic arm by means of the other robotic device attached at its end-effector. The joint error is fed into the CF pathway, the desired plan into the MF pathway; the DCNs modulate the anticipatory corrective torque.

Three paradigms were designed to mimic human learning observed in neurophysiological studies: (1) Eye Blinking Classical Conditioning (EBCC), which was tested both in computational simulations and in real robot, (2) Vestibulo-ocular Reflex (VOR), tested in real robot, and (3) upper-limb perturbed reaching, tested in real robot. The same cerebellar SNN was embedded into the controller with inputs and outputs customized for each specific protocol (Fig. 2).

#### EBCC in simulations and Associative Pavlovian task in real robot

During EBCC, pairs of a Conditioned Stimulus (CS: a stimulus that normally does not evoke the blink, like a tone) and an Unconditioned Stimulus (US: a stimulus, such as an air puff, that normally evokes the blink Unconditioned Response (UR)), presented at a constant Inter-Stimuli Interval (ISI), promote the acquisition of a Conditioned Response (CR), i.e. a blink response anticipating the US with its peak time-locked to the US onset. Once acquired this temporal association between stimuli, the CR is evoked by applying the CS alone, until a progressive extinction of that learned association takes place [40] (Fig. 2A).

The EBCC protocol consisted of a sequence of 400 repetitions where CS-US pairs were presented (acquisition phase) followed by a sequence of 200 CS-alone trials (extinction phase). For each repetition, the CS was generated as a random spike pattern on the 20 MFs, with an instantaneous spike probability equal to 5% (i.e. 50 Hz). The US was produced as a random spike pattern on the 24 IOs, with an instantaneous spike probability equal to 1% (i.e. 10 Hz), lasting 100 ms and ending together with CS (“delay-EBCC”).

In order to validate whether the MF input features and the GRs architecture were adequate to let the GR layer works as a not-recurrent state generator, a similarity index *C* was computed by measuring the correlation between each pair of granular layer states (i.e. pairs of time steps) within-trial [41], [29], [42]:
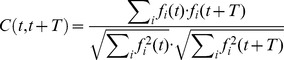
where *f_i_* corresponds to the instantaneous frequency of the *i-th* neuron (frequency measured within a 20-ms time window) and *C(t,t+T)* takes values from 0 to 1 (0 if the two activity vectors are complementary, 1 if they are identical).

The DCN spike patterns were decoded into analog signals at each time sample [36]: if a spike occurred, a fixed amount was added to the ongoing output, whereas if no spike occurred, a decay function was applied. Then, the output was averaged on a moving window of 100 samples (100 ms), then sent as motor command. This was the SNN output signal driving the behavioral outcomes. When it crossed a pre-set threshold equal to 20, a CR was generated.

Nucleo-olivary inhibition was introduced in the model: when a CR occurred, the US had a lower excitatory effect on the IOs, i.e. the spike probability was decreased to 0.5% (5 Hz). Indeed, the eyelid blink (CR) modifies the effect of the air-puff (US), since the noxiousness of the latter diminishes if the cornea is protected by the eyelid; however, the inhibition should not be complete, because perceiving the airpuff is needed to trigger the anticipatory action the next time the CS is perceived. Hence, the modulation of the IO firing probability is fundamental both to stabilize acquisition and to avoid overtraining [40], [14].

First of all, we focused on the setting of LTD and LTP parameters of the cerebellar model, by exploring the effects of different α and β on EBCC learning in computational simulations. As first step, a gross exploration of 25 combinations of α and β was performed, for each one repeating an EBCC test with 400 trials of acquisition and 200 trials of extinction. ISI was set to 300 ms, with CS lasted 400 ms and US 100 ms; each trial duration was 500 ms, whose last 100 ms were rest (all neurons silent). In each EBCC test, the acquisition capability and rate were quantified by the maximum output reached during the acquisition trials (maximum among the DCN within-trial maxima) and by the first trial during which the first CR was produced. As index of acquisition stability, the standard deviation of the DCN maxima, from the trial during which the first CR was generated and the last trial of acquisition (400^th^), was computed. Finally, the extinction capability was quantified by the maximum DCN output achieved within the last 10 trials of the extinction phase. Thus, the model tuning was performed so as the cerebellar controller would be able to (i) produce high DCN output, (ii) reach the CR threshold as fast as possible, (iii) minimize the variations during late acquisition, and (iv) minimize the DCN output at the end of extinction.

Afterwards, in the parameter sub-space meeting these requirements, a finer exploration was carried out, running EBCC simulations with further 100 combinations of α and β, and evaluating the same parameters as for the gross exploration. Finally, α and β have been set within a steady sub-space and kept for all the simulations and real-robot tasks.

In order to test the generalizability of the tuned cerebellar circuit, at least in computational simulations, other two EBCC tests with shorter and longer ISIs, within a physiologically effective range [43], [44], were carried out.

- ISI = 200 ms, with CS lasted 300 ms and US 100 ms; each trial lasted 400 ms, whose last 100 ms were rest (all neurons silent).
- ISI = 400 ms, with CS lasted 500 ms and US 100 ms; each trial lasted 600 ms, whose last 100 ms were rest (all neurons silent).

In real robot, the Pavlovian EBCC-like protocol was translated into a collision-avoidance task [28], [29], [14]. As for EBCC computational simulations, each test was made up of 400 trials of acquisition and 200 trials of extinction.

The robotic arm (Phantom Premium™) was moving on a pre-defined straight trajectory (using joint torques computed through a Proportional-Derivative feedback controller (PD), given the desired joint kinematics and the actual joint kinematics). During the acquisition phase, a fixed obstacle, attached to the other robotic device, was placed along that path (Fig. 2B). The CS was the visual stimulus indicating that the arm was approaching to the obstacle (“warning”), triggered by a threshold algorithm based on the distance between obstacle-vertex and robot end-effector (CS-threshold), detected by the tracking system; whereas the US was triggered by the collision event. The CS was carried by the MFs and the US by the CFs (Fig. 2C). During the extinction phase, the obstacle was removed and hence no US signal was fed into the circuit.

The CR generation was computed from the DCN firing rate, analogously to the output decoding approach described above for EBCC simulations. The UR was a stop reaction after the US-collision, achieved by a feedback control. The CR was a stop anticipating the US-collision, triggered by the cerebellar network. As in EBCC simulations, the nucleo-olivary inhibition was implemented: when a CR had been just produced during an acquisition trial, an inhibited US pattern was fed into the IOs; and the US onset was defined based on an estimated ISI, i.e. equal to the last one achieved when an actual US-collision occurred.

In order to validate the robustness and stability of the embedded cerebellar controller, different stimuli patterns, i.e. two CS-thresholds (CS-th_1_ 125 mm; CS-th_2_ 110 mm), were defined so as to generate two different ISIs (not perfectly repeatable across trials because of the actual robotic environment); for each CS-threshold, 12 tests were carried out.

For both simulated and real robot tests, the number of CRs and the anticipation of CRs with respect to US onset were computed. The CR number was the percentage of CR occurrence within consecutive blocks of 10 trials each. The CR anticipation was calculated as the time difference between the DCN activity causing a CR and the US onset.

#### VOR in real robot

The VOR consists of eye movements stabilizing images on the retina during head motion, and its tuning is ascribed mainly to the cerebellar flocculus [45] (Fig. 2D).

The VOR protocol was reproduced in real robot by using the 2^nd^ joint as the head, on which a desired joint displacement was imposed, and the 3^rd^ joint as the eye motion driven only by the cerebellar SNN. The set-up was arranged so that the two involved joints (2^nd^ and 3^rd^) moved on a horizontal plane (Fig. 2E). The Head Rotation (HR) generating the vestibular input was provided to the 20 MFs exploiting Radial Basis Functions (RBFs) to transform the analog angular time-profile into spike patterns [38]; the Gaussian RBF centers were equally distributed in the angular range. The visual error, thanks to the tracking system, was computed as the disalignment angle between the actual gaze direction, i.e. the orientation of the second link of the robot, and the desired one aligned with the object to be fixed; this analog error was translated into irregular spike patterns on the group of 12 IOs corresponding to the actual error sign, through a probabilistic Poisson process where the instantaneous error signal was compared to a number between 0 and 1 randomly generated each time [46]. The firing rate of the DCN spiking activity was proportionally translated into a net torque on the 3^rd^ joint, positive or negative depending on the error sign at each time sample [36] (Fig. 2F).

Similarly to the Pavlovian task, the protocol consisted of a sequence of 400 repetitions where a head turn was imposed and the target object was fixed, followed by 200 repetitions with the same head turn but with the target moving in the same direction as the head, thus requiring a strong VOR gain-down [47].

In order to validate the robustness and stability of the embedded cerebellar controller, different stimuli patterns, i.e. two Head Rotations (HR_1_ from 0° to 30° and back to 0° in 2 seconds, HR_2_ from 0° to 20° and back to 0° in 2 seconds) were imposed; each trial lasted 3 seconds: 2 seconds of movement and 1 second of rest. For each HR, 12 tests were carried out.

Root Mean Square (RMS) of gaze direction error was computed.

#### Perturbed reaching in real robot

Force field compensation during arm reaching movements critically depends on the cerebellum, since it acts as a comparator of predicted and perceived state of the limb, adapting to new dynamics [48] (Fig. 2G).

The arm desired kinematics was defined on the 3 DoFs in joint-space, and achieved thanks to a simple PD feedback controller. After 50 repetitions without any external disturbance, a viscous force field was introduced, as *F(t) = c·v(t)*; where *F(t)* is the Cartesian force produced by the secondary robot on *z* direction perpendicular to the trajectory of the moving end-effector, *c* is the constant field strength, *v* is the *x*-component of the end-effector Cartesian velocity, where the *x* direction is the trajectory direction in the robot reference frame. The viscous force field pushed the moving end-effector, thus deviating significantly the trajectory by perturbing mostly the 3^rd^ joint (Fig. 2H).

Similarly to the previously described robotic tasks, after a baseline phase of 50 trials, the disturbing force lasted 400 repetitions (acquisition phase), followed by 200 extinction trials in which the force field was removed. Analogously to the encoding and decoding strategies used for the VOR protocol, the 20 MFs were fed with the 3^rd^ desired angle translated into spikes by RBFs, while the 3^rd^ joint error was translated by a Poisson approach into spikes to the 12 IOs of the corresponding sign. The DCN firing rate was proportionally translated into a net torque on the 3^rd^ joint and added to the PD feedback torque (Fig. 2I).

In order to validate the robustness and stability of the embedded cerebellar controller, different perturbations, i.e. two Force Field constants (c_1_ = 0.0040 kg/s, c_2_ = 0.0033 kg/s), were imposed during the forward phase of the trajectory, lasting 1 second. Then, 3 seconds were used just to go back and recover the initial configuration. For each force field, 12 tests were carried out.

RMS of the 3^rd^ joint deviation was computed.

## Results

A realistic SNN of the cerebellum [38] was customized, embedded into a real-time system controller and integrated into a sensorized robotic platform. In order to test the generalized learning and control capabilities of this neurorobot, we have designed prototypical human-like sensorimotor tasks, addressing the main aspects of learning and control: (i) associative learning of a well-timed stimuli association (EBCC), (ii) combined learning of timing and gain in a closed-loop reflex movement (VOR), (iii) combined learning of timing and gain in a closed-loop voluntary limb movement (force-field reaching). By varying stimuli and perturbations patterns, control robustness and generalizability, driven by implicit spiking dynamics of the same cerebellar model, have been investigated.

### Cerebellar SNN tuning

**Figure 3:**
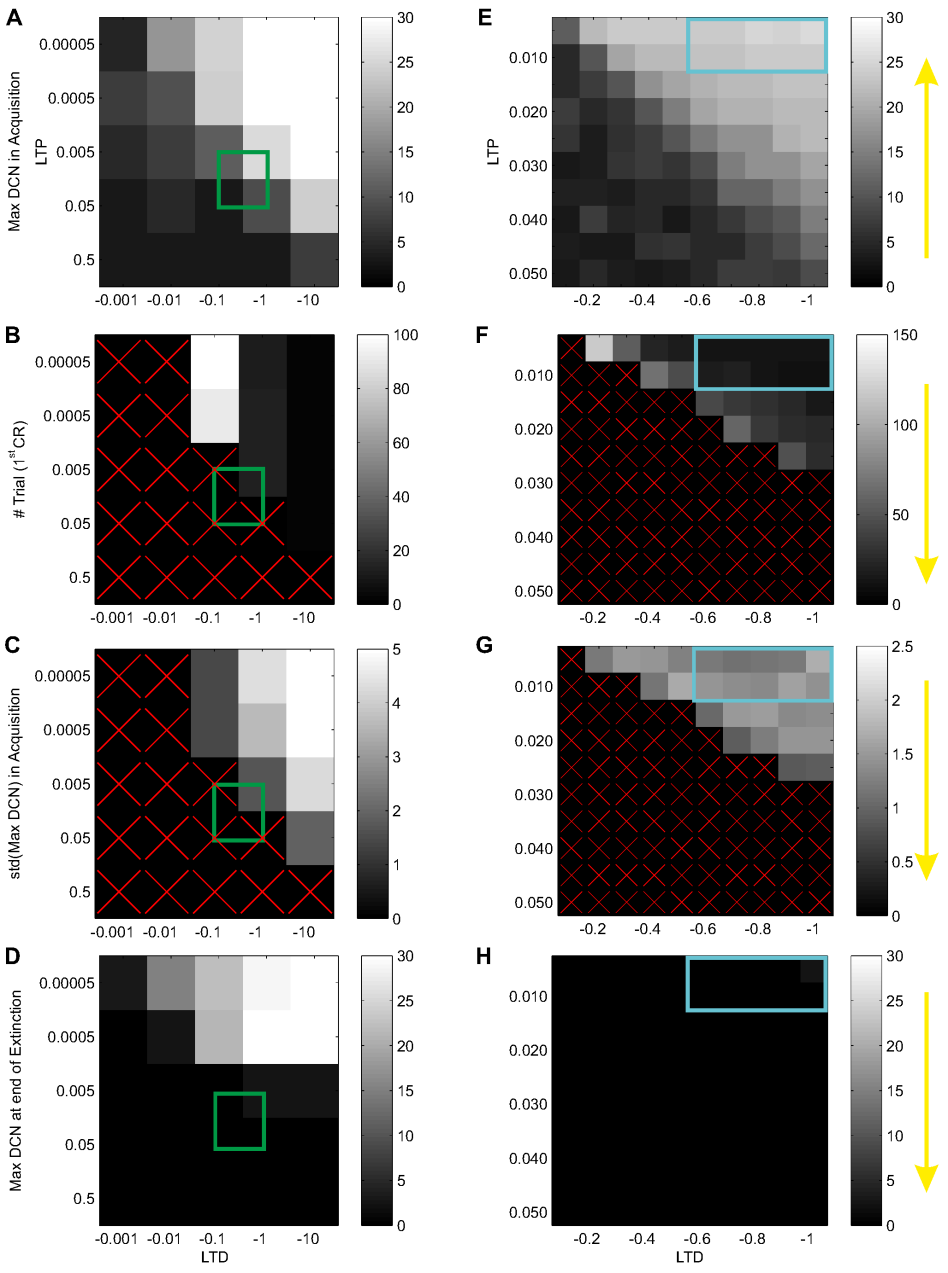
Cerebellar SNN tuning by EBCC simulations. Exploration of LTD and LTP parameters in EBCC tests (400 trials of acquisition and 200 trials of extinction). A gross exploration (first column) with 25 combinations (centers of each pixel) and then a finer exploration (second column) with 100 combinations (i.e. pixels) of the parameter space were carried out. The impacts on learning were quantified by the maximum of the DCN within-trial maxima (A and E), by the number of the first trial when CR threshold was overcome (B and F), by the standard deviation of the DCN maxima during late acquisition (C and G), and by the DCN activity at the end of extinction (D and H). Yellow arrows indicate the optimal directions of these indexes. Green squares on the first column represent the gross area selected for the finer exploration. The light blue squares in the second column represent the optimal area within the LTD and LTP parameters have been chosen. Red cross within pixels means no results came out with that combination of parameters, relative to the specific index.

From the gross exploration of LTD and LTP parameters, through a series of EBCC simulations, we identified an acceptable parameter subspace, where the SNN controller was able (i) to exploit the DCN firing activity range, reaching a maximum output close to the DCN maximum firing rate, (ii) to reach the CR threshold as fast as possible during the acquisition trials, (iii) to minimize the variations during late acquisition, and (iv) to minimize the DCN output at the end of extinction, close to silent DCN status (Fig. 3A, B, C and D). Then, with a higher resolution exploration, we evaluated the same indexes in further EBCC simulations (Fig. 3E, F, G and H). The best performance was figured out with α = 0.005 and β = −1. Anyway, in the neighborhood, the behavioral outcomes were comparable and meaningful, thus demonstrating a stable and reliable performance of the SNN controller around the chosen parameters.

### EBCC simulations

The EBCC is a paradigm of associative temporal learning [49], used as a prototype to investigate the cerebellar function. The cerebellum learns to generate a CR time-locked and anticipated with respect to the US, with the CR maximum occurring at the time when the US is expected [40].

**Figure 4:**
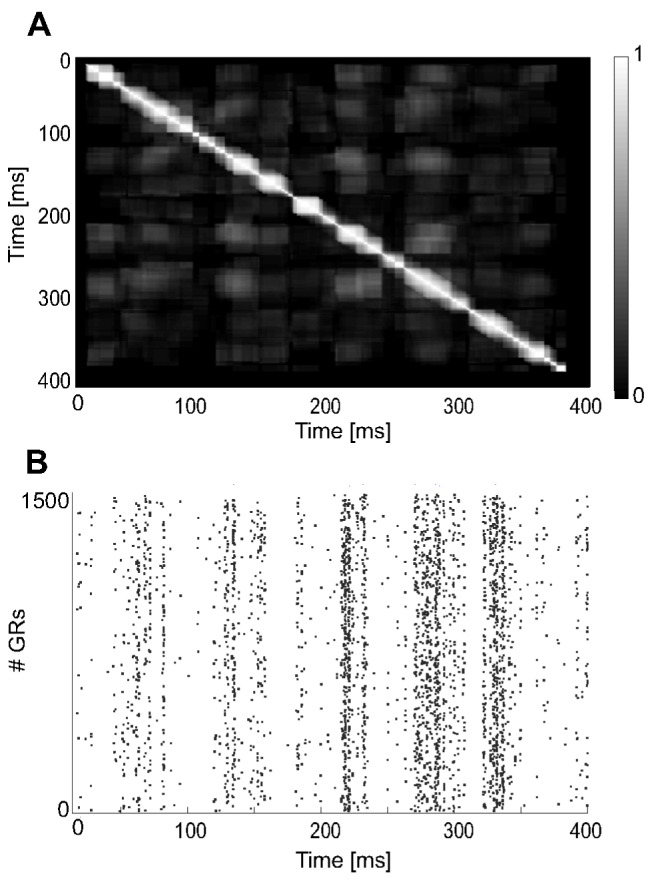
Granular layer: spatio-temporal activity. (A) similarity indexes between pairs of instantaneous patterns in the GR layer during a 400-ms CS sent as a 50 Hz random activity on the 20 MFs. The values of indexes are represented in grey scale; black 0, white 1. The darker the matrix is, the better uncorrelated activity patterns are. (B) raster plot of the 1500 GR cells during the CS.

The CS-related mossy fiber spike pattern was expanded into the granular layer, robustly operating as not-recurrent state generator, as validated by the GR layer similarity matrix during the 400 ms of CS-related MF activity of one acquisition trial (Fig. 4). *C(t,t+T)* was 1 when *T* = 0 because of the trivial identity, while it monotonically decreased as *T* increased, with a mean value (excluding the main diagonal) equal to 0.126. The granular layer hence represented univocally the passage of time and the system state.

**Figure 5:**
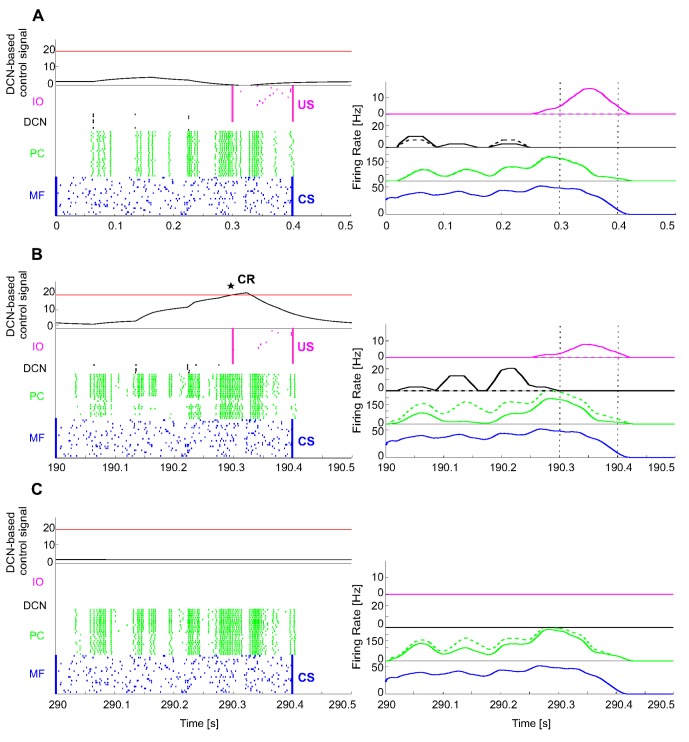
EBCC in simulations: SNN working. The first test of delay-EBCC was implemented with ISI = 300 ms, 400-ms CS and 100-ms US; each repetition lasted 500 ms. The protocol consisted of 400 repetitions of acquisition (CS-US pairs) and 200 of extinction (CS-alone). On the left column, the raster plots of the network activity (excluding the 1500 GRs) of three trials in different phases of the learning process: early acquisition, late acquisition and late extinction. Aligned on the right, the activity of each cell population as mean of the active cells' instantaneous firing rates (spike counting within a mobile 25-ms time window and then 50-ms smoothing). In all the trials, the CS-related MF spike pattern was equal to 39±8 Hz. The IOs showed a firing rate of 10±3 Hz in the trials where no response was generated ahead of the US onset (A); whereas, the IO firing rate was reduced to 5±2 Hz when a CR anticipated the US onset (B). (A) At the beginning of the acquisition (1^st^ repetition), PCs were spontaneously active, with an overall firing rate during the CS of 81±49 Hz, supplying tonic inhibition to the DCNs. (B) The activity pattern of Purkinje cells was altered during conditioning, reducing the firing rate in a time-dependent manner (68±58 Hz), thanks to a temporal-specific LTD at PF-PC connections. Consequently the DCN activity increased (8±6 Hz), overcoming the threshold before the US onset. Hence, a CR was produced (black star) (380^th^ repetition). (C) After some trials in which the network output still produced CRs even if only CS was presented, a complete extinction, driven by the LTP mechanism, re-increased the PCs activity and hence re-inhibited the DCNs (580^th^ repetition).

From GRs, activity was transmitted to PCs. MF activity also excited the DCNs. The US-related spike pattern from IOs reached the PCs, which in turn inhibited the DCNs. During the 400 ms of CS, the mossy fibers had a mean firing rate of 39±8 Hz, while the IOs, during the 100 ms of US, had a mean firing rate of 10±3 Hz. While the mossy fibers always conveyed the same pattern, the IO firing rate was inhibited to 5±2 Hz when DCNs had acquired predictive motor responses (CRs). At the beginning of EBCC acquisition (Fig. 5A), PCs were spontaneously active, with a firing rate of 81±49 Hz and supplied tonic inhibition to the DCNs. The activity pattern of Purkinje cells changed during conditioning: their firing rate decreased along with the development of PF-PC LTD. During the CS, the PC firing rate was reduced to 68±58 Hz and the firing DCNs passed from 4±3 to 8±6 Hz, producing CRs (Fig. 5B). Then, for some trials, even if only CS was presented, the network output still produced CRs, until a complete extinction was driven by PF-PC LTP, which raised PCs activity and hence re-inhibited the DCNs (Fig. 5C).

**Figure 6:**
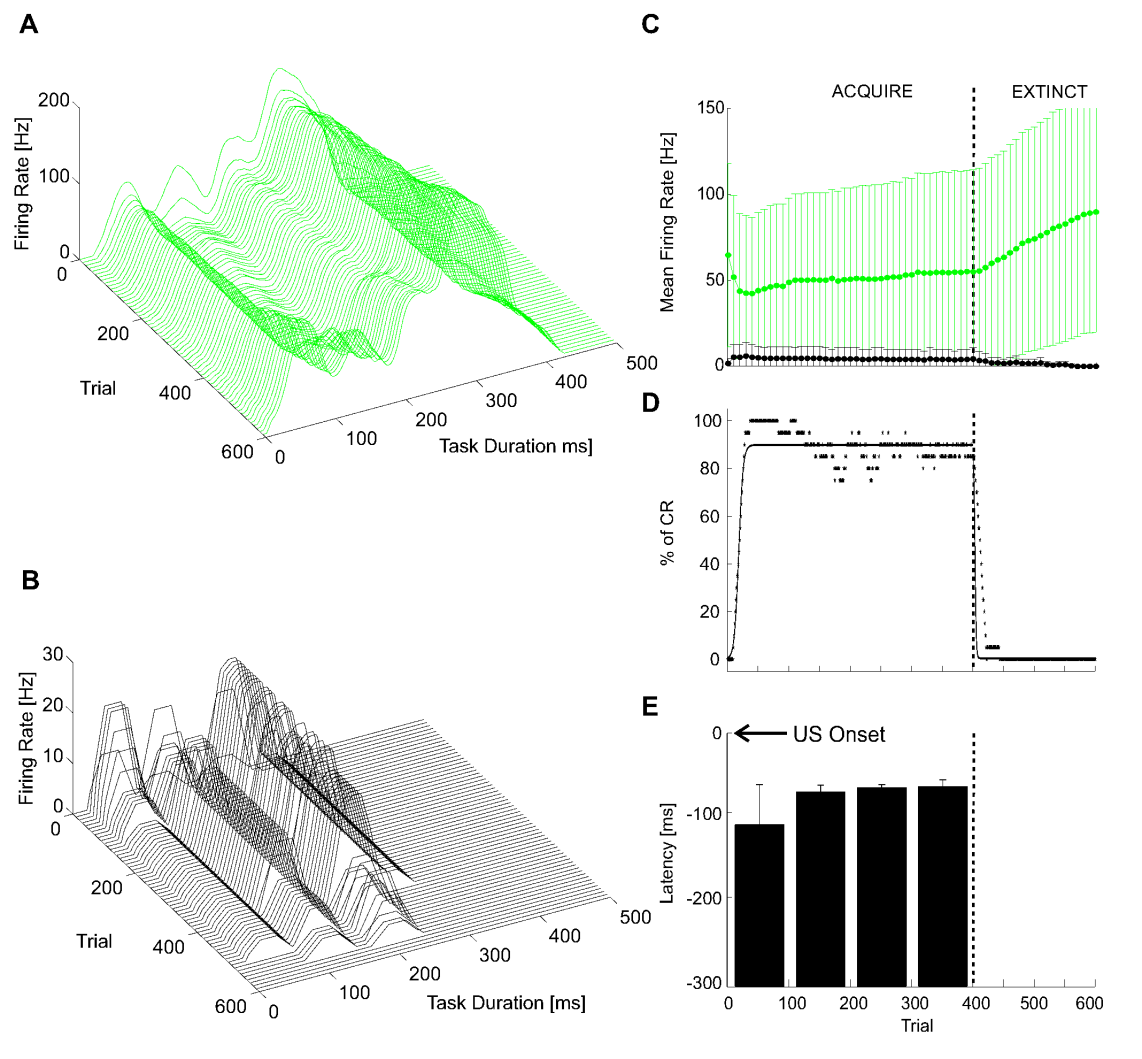
EBCC in simulations: motor response generation. 3D plots of PC activity (A) and of DCN activity (B), along time and trials (one each 10 repetitions, for picture clarity). The activity is computed as firing rate in a mobile 25-ms time window, averaged across all cells of each population. (C) PC firing rate, averaged within each trial (during CS-related MF excitation). The whiskers represent the standard deviations. One each 10 repetitions is depicted for picture clarity. (D) Number of CRs (%) along acquisition and extinction trials, computed as percentage number of CR occurrence within consecutive blocks of 10 trials each. Both acquisition and extinction phases were fitted by the best least-squares sigmoid fitting curves, i.e. the ones minimizing the residual error (Root mean square error: 6.091% for the acquisition phase, 5.758% for the extinction one). The vertical dashed line highlights the shift between acquisition and extinction phases. (E) CR anticipation, computed as mean within blocks of 100 trials each. The whiskers represent the standard deviations of the CR latency within each block. The vertical dashed line highlights the shift between acquisition and extinction phases.

An extended representation of the whole family of responses in PCs and DCNs (Fig. 6A and 6B) shows that, during learning, PC firing rate decreased while DCN firing rate increased progressively (Fig. 6C). During extinction the firing rates tended to recover to their initial values. Learning and extinction proceeded along a sigmoidal time-course (R^2^ = 0.885 for the acquisition curve and R^2^ = 0.893 for the extinction curve). At the end of the learning process, a mean CR occurrence of 88±5% (averaged over the last 300 acquisition trials) was achieved, comparable to the level of CRs acquired in neurophysiological experiments [50] (Fig. 6D). Moreover, at the end of the learning process, CRs were closer to US onset than at the beginning; the average anticipation value during the whole acquisition phase was 77±30 ms (Fig. 6E).

**Figure 7:**
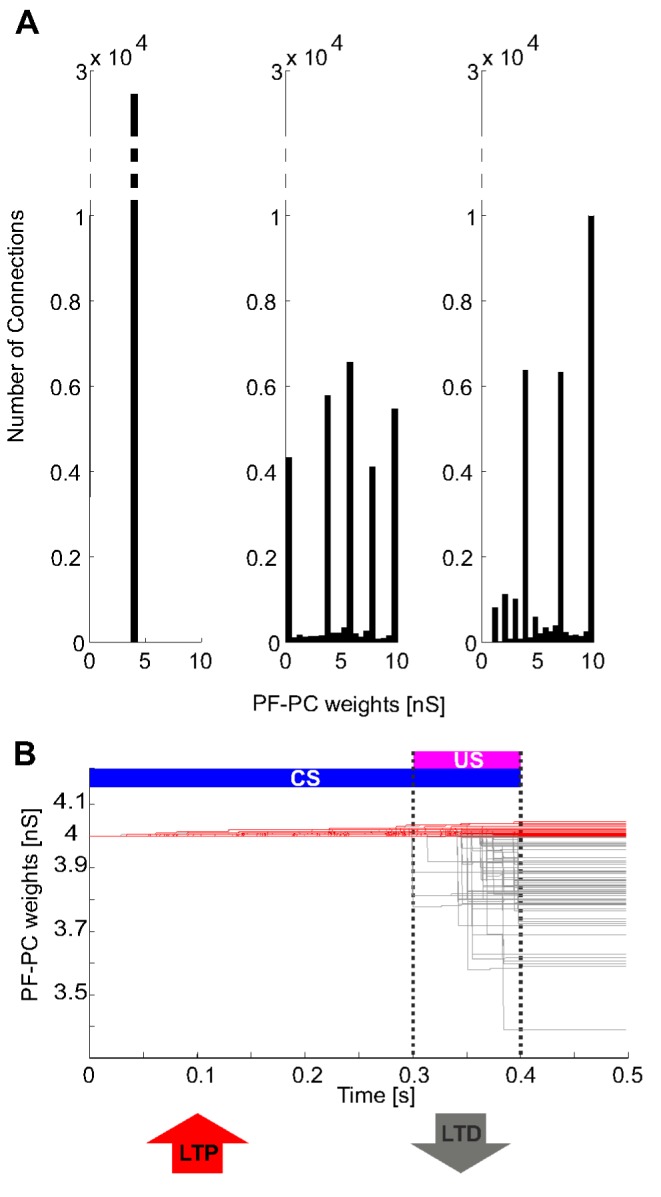
Plasticity. (A) histograms of the PF-PC weights at the end of three trials (1^st^, 380^th^, 580^th^, as in Fig. 5). All weights were initialized at 4 nS. (B) blow-up of PF-PC synaptic weights, depicted one each 100 (288 lines) for picture clarity, within one repetition (the 1^st^ trial, from 0.0 to 0.5 s), in order to show the time-dependent changes of each weight. In red, the weights underwent basically a LTP during the test, while in grey the overall depressed synapses (LTD).

In the model, there were 28870 plastic synapses (around 80% of #GRs·#PCs), some undergoing LTP and others LTD. From an intermediate and neutral initial value, the synapses involved into the state representations during ISI moved towards higher weight values; while, the PF-PC synapses mostly activated into the state representations associated with the incoming US depressed their weights towards zero (Fig. 7A). Indeed, within single trials, during the first 300 ms (i.e. with only CS-related mossy fiber signals), a slight LTP occurred for all synapses since parallel fibers carried to PCs a state-related activity without any associated activity coming from the IOs. When US arrived, a robust LTD took place on the active PF-PC synapses (Fig. 7B).

**Figure 8:**
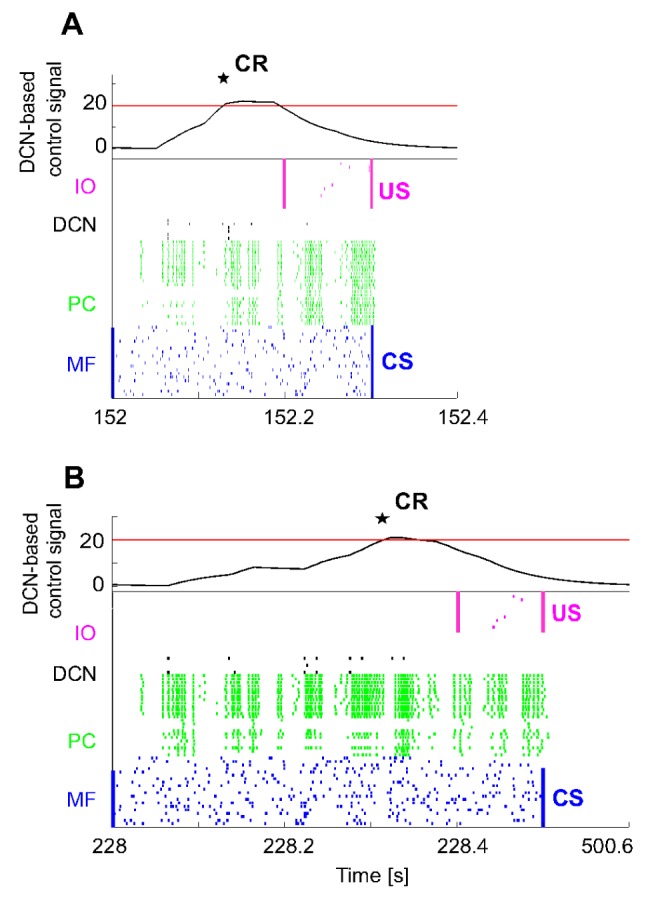
EBCC in simulations: different ISIs. One representative trial for each of the other two tests of EBCC, taken in late acquisition when a stable CR generation was achieved (380^th^ trial). (A) ISI = 200 ms (B) ISI = 400 ms. The cerebellar SNN was able to associate different combinations of stimuli.

Even with different ISIs, the cerebellar SNN learned to effectively produce CRs time-locked to the US onset (Fig. 8). In the case of a longer ISI, the CR occurrence was 93±8% (averaged over the last 300 acquisition trials) and the CR anticipation was 170±134 ms; whereas with a shorter ISI, the CR occurrence was 95±5% and the CR anticipation was 57±10 ms. It is consistent with a positive correlation between ISI and CR anticipation [51].

### Associative Pavlovian task in real robot

**Figure 9:**
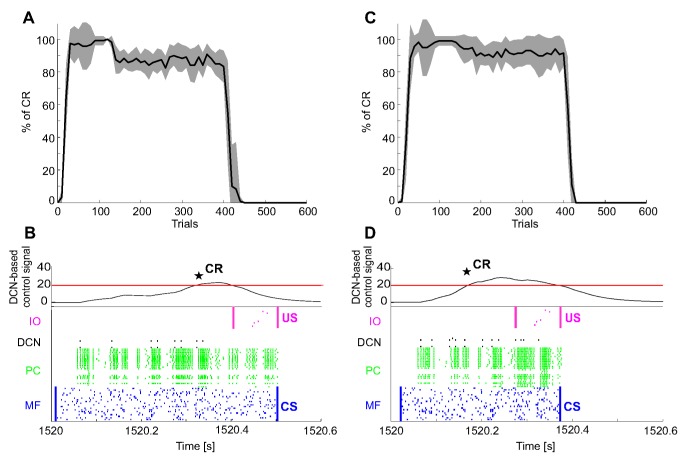
Associative Pavlovian task in real robot. (A) and (C) Number of CRs (%) along trials (400 acquisition trials and 200 extinction trials), computed as percentage number of CR occurrence within blocks of 10 trials each, for real robot tests with ISI_1_ and ISI_2_. For each ISI, the black curve is the average on the 12 tests, and the grey area is the standard deviation. In spite of the uncertainty and variability introduced by the direct interaction with a real environment, the cerebellar SNN was able to progressively learn a CR anticipating the US and to extinguish it. (B) and (D) Raster plot of the recorded network activity (excluding the 1500 GRs), in late acquisition when a stable CR generation was achieved (380^th^ repetition, from 1520 s to 1520.6 s). The learning process induced a temporal-specific LTD at PF-PC connections, thus reducing the PC activity and consequently increasing the DCN activity, which overcame the threshold just before the US onset. Hence, a CR was detected (black star).

The repeatability observed in EBCC was not guaranteed when Pavlovian conditioning was implemented as a collision-avoidance task performed by a real robot, because of the presence of uncontrolled and noisy real-world conditions (Fig. 9). Indeed, the ISIs were not constant; the mean ISI_1_ (with CS-th_1_) across all the acquisition trials of the 12 tests was 401.2±67.7 ms; while the mean ISI_2_ (with CS-th_2_) was 326.9±78.7 ms. It was due to the refresh of the tracking system and to some trajectory variability across repetitions due to inertial components of the robot. However, the cerebellar SNN was able to progressively produce predictive CRs, slightly less stable than in EBCC computational simulations. At the beginning of acquisition, no output was produced by DCNs, resulting in collisions between the robot end-effector and the obstacle. During learning, CRs (anticipated stop responses) were produced, thus preventing collisions. At the end of the training phase, the CR rate with ISI_1_ was 92±10% and the CR anticipation was 141±133 ms (averaged over the last 300 acquisition trials of the 12 tests); with ISI_2_, the CR rate was 92±11% and the CR anticipation was 100±68 ms (Fig. 9A and C). The firing patterns in the SNN controller showed a behavior similar to the EBCC computational simulations (Fig. 9B and D). Thus, although with the real robot the SNN controller had to cope with additional sources of instability, a CR learning similar to the simulations was generated, even if with a higher standard deviation.

### VOR in real robot

The VOR is a paradigm of time-dependent gain learning in reflex movements, in which phase and gain of eye movements have to be finely tuned in order to obtain image stabilization during head rotation. For both test conditions, the maximum HR came out not perfectly constant, both across trials of the same test and across the 12 tests (HR_1_ = 30.8±1.3°; HR_2_ = 20.3±0.6°).

**Figure 10:**
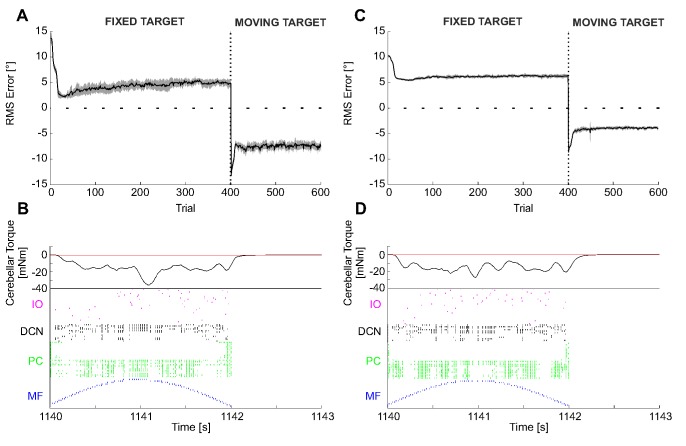
VOR in real robot. (A) and (C) Gaze angular error (RMS within each trial) during 400 repetitions with fixed target and 200 repetitions with moving target, which required a strong reduction of the previously learned VOR. For each HR (HR_1_ and HR_2_), the curve is the average on the 12 tests, and the area is the standard deviation. (B) and (D) Raster plot of the recorded network activity (excluding the 1500 GRs), in one repetition (380^th^ repetition), where the acquisition had been completed, characterized by a stable and effective compensatory eye movement. There was still an error (negative), but the IOs fired at a very low rate. The head rotation on the 2^nd^ joint (vestibular stimulus) was decoded by the MFs through RBFs. The DCN activity was significant for the negative DCNs, which produced a negative torque, applied on the third joint; thus, it produced an eye turn, with the same shape of the head turn but the opposite sign, and with an intensity peak of 36 mNm for HR_1_ and 27 mNm for HR_2_. Note: there was no MF and PC activity during the last second because that part of the repetition was only necessary to steadily bring back the robot to its initial position, without any cerebellar activation.

During the head movement, the gaze deviated from target. At beginning of learning, the RMS gaze error was up to 13.7°±0.9° and 10.3°±0.2° for HR_1_ and HR_2_, respectively. During learning, the cerebellar SNN tuned eye motion in order to continuously compensate head rotation. At the end of training, the gaze RMS error was reduced to 4.6°±0.4° and 6.2°±0.1° for HR_1_ and HR_2_, respectively (averaged over the last 300 acquisition trials of the 12 tests). After allowing the system to stabilize, the target began to move in the same direction as the head. This caused an initial overcompensation determining an absolute RMS gaze error of 13.2°±1° and 8.6°±0.2°, for HR_1_ and HR_2_, respectively (at the first extinction trial, i.e. moving target, averaging the 12 tests), with a mean gaze error value of the opposite sign. The VOR was then re-modulated dropping the absolute RMS error back to 7.5°±0.3° and 3.9°±0.1° for HR_1_ and HR_2_, respectively (averaged over the last 10 extinction trials of the 12 tests) (Fig. 10A and C).

The SNN did not perfectly achieve the ideal zeroing of the gaze error. It is due to the not repeatability in time and space across trials for uncertainty in the motion and sensory recordings of the real low friction robot used in the tests. Moreover, the chosen cerebellar parameters were optimized on EBCC simulations; a partially task-dependent tuning could enhance the performance, although fast and stable acquisition and extinction were robustly expressed also in this task.

The mossy fibers encoded the head angle, the climbing fibers encoded a negative error, the PC firing rate decreased due to LTD letting the corresponding DCNs to proportionally increase their firing rate based on the present head rotation state. The DCN firing rate corresponded to an eye torque (amplified by a constant gain equal to 0.5), able to compensate for head rotation (Fig. 10B and D).

### Perturbed reaching in real robot

The perturbed reaching is a paradigm of time-dependent gain learning in voluntary multi-joint movements. The cerebellum produces an accurate compensation to external force. It is added to the feedback motor commands, which are not able to learn and anticipate the perturbation effects. For both test conditions, the maximum external force came out not perfectly constant, both across trials of the same test and across the 12 tests (1.25±0.03 N for c_1_; 0.92±0.02 N for c_2_).

**Figure 11:**
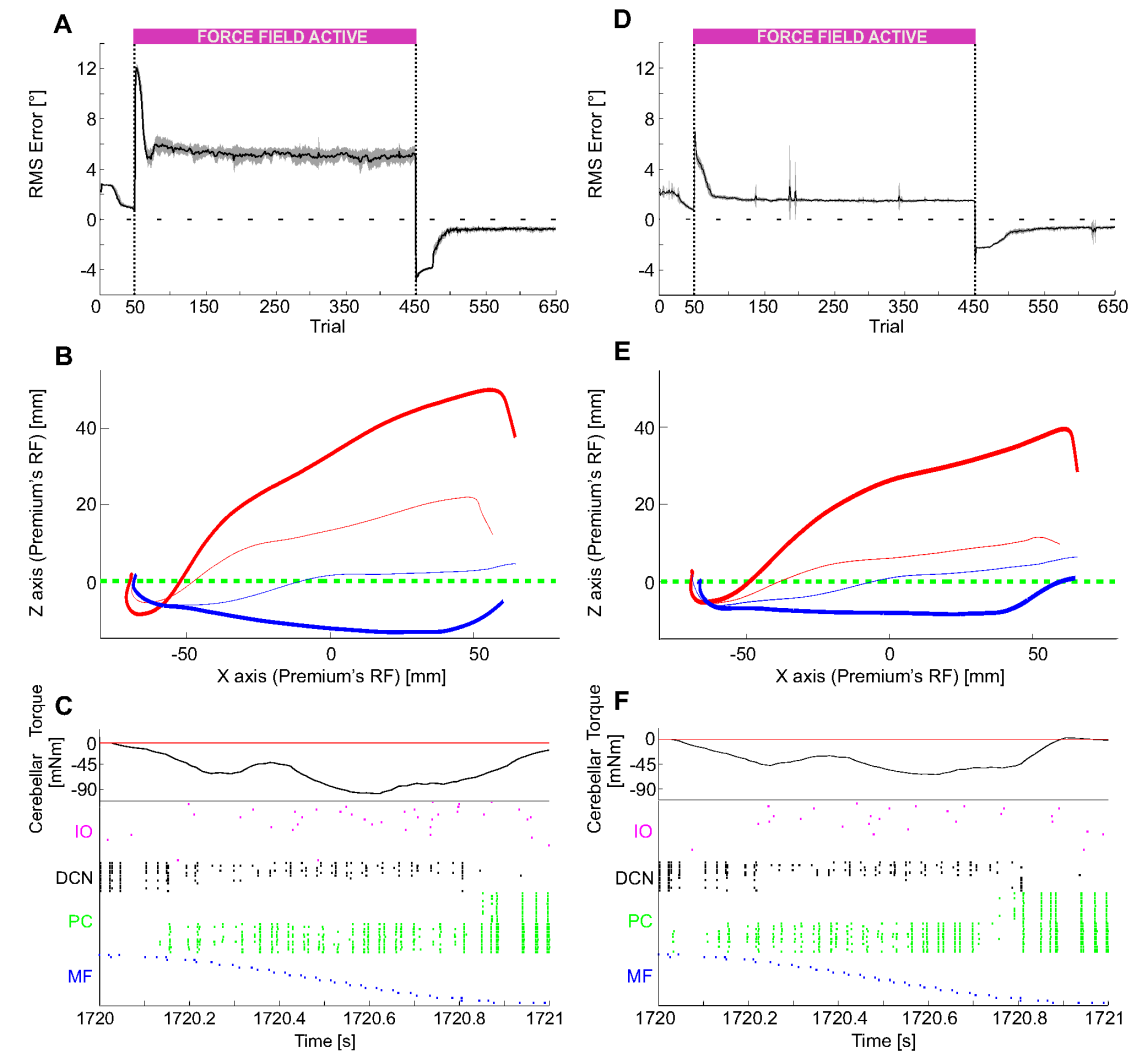
Perturbed reaching in real robot. (A) and (D) Joint angular error (RMS within each trial) during the 650 repetitions: 50 trials without force field, then 400 with a viscous force field (force field c_1_ and force field c_2_) and the last 200 again with null force field. The last phase clearly shows the after-effect phenomenon, occurring when the perturbation was suddenly canceled after a complete acquisition. For each force field, the curve is the average on the 12 tests, and the area is the standard deviation. (B) and (E) For each force field, Cartesian trajectories on the xz plane in some representative trials: ideal trajectory in dashed green, 1^st^ perturbed trial (51^st^) in thick red, last perturbed trial (450^th^) in thin red, 1^st^ re-unperturbed trial (451^st^) in thick blue, last trial (650^th^) in thin blue. (C) and (F) Raster plot of the recorded network activity (excluding the 1500 GRs), in one repetition (430^th^ repetition), where the acquisition had been completed, characterized by a stable compensation of the constant external perturbation. There was still an error (negative and positive), but the IOs fired at a very low rate. The DCN activity was evident almost only on the negative DCNs, which produced a negative corrective torque on the third joint, able to counterbalance the viscous force fields.

During the first 50 trials without any external force disturbance, the cerebellum action was just a refinement keeping the deviation (proprioceptive-like error, or joint error) near to zero (1.8±0.8° and 1.7±0.5° for c_1_ and c_2_, respectively, averaged over the first 50 trials of the 12 tests). Afterwards, when the force field was suddenly activated, the 3^rd^ joint motion did not compensate for it, bringing the 3^rd^ joint RMS error to 10.6°±0.3° for c_1_ and to 6.9°±0.3° for c_2,_ respectively (at the first trial of the activated force field, averaging the 12 tests). The mossy fibers encoded the desired angle of the 3^rd^ joint, the climbing fibers encoded alternatively a positive or negative error, the PC firing activity decreased due to ensuing LTD, letting the corresponding DCNs to proportionally increase their firing rate, which was translated into a torque sent on the 3^rd^ joint, amplified by a constant gain equal to 1.5 and to 1 for c_1_ and c_2_, respectively. During learning, the SNN tuned the 3^rd^ joint torque in order to continuously compensate for the force field. After adaptation, the RMS error decreased to 5.1°±0.2° and 1.5°±0.1° for c_1_ and c_2_, respectively (averaged over the last 300 acquisition trials of the 12 tests). Then, the force was removed yielding an overcompensation and causing a rebound of RMS error to 4.9°±0.2° and 3.3°±0.1°, for c_1_ and c_2_, respectively (at the first trial of force field cancelation, averaging the 12 tests), with a mean gaze error value of the opposite sign. The corrective motor command was eventually re-modulated causing the RMS error to drop back to 0.7°±0.1° and 0.6°±0.1° for c_1_ and c_2_, respectively (averaged over the last 10 extinction trials of the 12 tests) (Fig. 11).

## Discussion

The main result in this paper is that a SNN of the cerebellum embedded into an appropriate control system can be successfully used to control a robot. As the modular structure of the real cerebellum, the same principles, embedded into the cerebellar microcircuit model, were applied to a variety of tasks with different nature, calling for complex timing and movement patterns. The cerebellar SNN learned to adjust timing and gain of the motor responses by predictively tuning its output to different input stimuli. Thus, the cerebellar SNN operated as a spiking adaptive forward controller. As a whole, the system demonstrated the main abstract functions ascribed to the cerebellum - timing, prediction and learning [52], [2], [53] - and allowed their analysis to be performed in terms of spike discharge in the different network components.

### The SNN operates as a generalized adaptive controller in real robot

The SNN processed mossy fiber inputs as arbitrary contextual signals, irrespective of whether they conveyed a tone, a vestibular stimulus or the position of a limb. The climbing fiber inputs conveyed the need for the cerebellar output to be greater at a particular time. The SNN accounted with high precision for the temporal shift between the information travelling in the MF and CF pathways. The issue of temporal credit assignment was faced through the eligibility trace embedded into the learning rule.

In all the tested sensorimotor tasks, the cerebellar SNN worked as a forward controller, i.e. able to provide motor commands in a predictive manner, progressively learning how to cope with the incoming sensory perturbations, along repetitions of task in procedural learning. The predictive function was also demonstrated by the after-effects occurring transiently when the external stimulus was suddenly changed after a stable behavior was achieved. The after-effect was evident in early extinction phases, (i) in the EBCC, when CRs were elicited by the CS alone, (ii) in the VOR, when an eye overcompensation was produced while the target started to move, and (iii) in the reaching task, when an overestimated torque was applied at the previously perturbed joint after the force-field was canceled. These over-actions of the cerebellar controller were rapidly re-modulated based on the new input configurations.

The here reproduced adaptation process and fast extinction reflect the known disparity in rates for adaptation and for abolishing the after-effects. Extinction was achieved without returning to the original configuration of weights. Such a configuration might be closer in weight space to the weight configuration that triggers the acquired responses, for which, at reacquisition, returning to this configuration might be achieved faster [14].

Being characterized by realistic architecture and spiking signals, our cerebellar SNN drove adaptive behaviors analogous to those found in humans in different closed-loop sensorimotor tasks. (i) In the EBCC-like, the robot achieved CR levels and anticipation values similar to those found in humans (who showed CR values of 72±14% and anticipation values of 140±30 ms with 400-ms ISI [50], [54]). It should also be noted that, as well as in humans, the ratio between ISI and anticipation tended to remain constant [55]. (ii) In the VOR, the SNN-driven overall behavior was comparable with neurophysiological studies focused on visual-vestibular training [56], [57], [58]. (iii) In the perturbed reaching, the robot behavior was comparable to neurophysiological studies in which healthy subjects performed a planar reaching protocol at natural speed perturbed by a viscous force field [59].

### Firing patterns of the cerebellar SNN

All learning paradigms were driven by the same network, designed with cell amounts of each population and ratios of connectivity as in real cerebellum, working with firing rates similar to those of the real cerebellar cell populations and with stable LTD and LTP rates of the plasticity mechanisms.

The crucial position of the cerebellum in the brain and its involvement in sensorimotor and cognitive processing make it an ideal structure for investigating the role of neural plasticity in learning [4]. The present spiking forward controller operated by regulating the firing pattern in DCN neurons under PC control [60]. Along learning, the response of PCs to MF inputs decreased and this increased the discharge in DCN neurons. The process was better exemplified in the adaptation of the EBCC, in which a precise time relationship between the events can be established. Since the DCN spike pattern changes occurred before the US arrival, the DCN discharge accurately predicted the US and therefore could facilitate the release of an anticipatory behavioral response. At the same time, the IO signal carrying US decreased. The prediction of a noxious stimulus triggers an anticipatory motor command. The inhibition mechanism of the IOs by the DCNs implies to translate the motor command into a sensory prediction signal, allowing a single cerebellar area to simultaneously tackle both motor execution and sensory prediction [14].

The generalizability of the learning model is demonstrated by the applicability of the same structural and functional principles of the cerebellar SNN to different operative conditions. As in Yamazaki's work using computational models [24], the granular layer was able to recode MF signals into a high-dimensional sparse spatiotemporal spike pattern, irrespective of whether mossy fibers conveyed constant signals (e.g. during the CS) or time-varying signals (e.g. during head rotation or arm angular displacement). A structural variant of the SNN used for the EBCC task only, both in simulations and in real robot tests, was the inhibitory projection from cerebellar nuclei to IOs. Indeed, in VOR and perturbed reaching (or Optokinetic reflex in [24]), image slip or joint error gradually decreased during training and this naturally diminished the CF signal to PCs without the need of any additional DCN-IO inhibitory mechanism.

In the cerebellar SNN, the parameter correlating response patterns to behavior was the average firing frequency. The effectiveness of SNN firing rates in controlling the robotic learning could be taken as an indication that rate coding is indeed appropriate to regulate sensorimotor activity, in line with the observation that frequency may be superior to delay (or latency) for accurately coding sensory inputs and motor outputs [26]. However, the biological reality is more complex. The cerebellum is endowed with mechanisms both tuning the composition and delay of well-timed spike bursts in the granular layer and modulating tonic firing discharge in Purkinje cells and DCN cells [61]. A simple hypothesis is that additional time-dependent mechanisms would further improve the cerebellar SNN performance and accuracy. A hint on how these different mechanisms could combine in optimizing cerebellar learning and control could emerge from the implementation of more realistic neurons and higher order firing pattern statistics (including millisecond-scale correlations and bursting) in the SNN (e.g. see [62] [63]).

Although, in the present SNN, all neurons were simple leaky Integrate&Fire and the architecture of the network was scaled and simplified, multiple associative behaviors could be reliably learned and processed, by neurophysiological firing rates of the different cell populations. Moreover, a single elementary site of plasticity was sufficient to drive learning.

The present SNN was constructed using a minimal structure to let the underlying operative principles to emerge. As expected, this strategy did not allow a full reproduction of the corresponding human behaviors. This suggests that the complex properties of biological firing patterns and local connectivity are required for higher order processing of multimodal firing patterns integrated into response cycles, as it occurs during complex multi-joint coordinated motor activities under control of cortical rhythmic discharges [51]. The neurorobot attained a stable human-like response level with also a human-like rate in all the tasks. Hence, the embedded computational model can acquire correct responses, even with the IOs sustaining a physiologically correct low rate of activity, without slowing down the learning processes. However, the accuracy was not always maximized, leaving not negligible residual errors. The model with one plasticity site, tuned only once and applied in multiple and completely different tasks, is able to produce a behavior as trade-off between rate, accuracy and versatility. Distributed plasticity sites could equip the model with a better capability to effectively adapt to any dynamic ranges of stimuli, operating as adaptive gain controllers. Furthermore, the SNN started from a naïve state, but finer circuit mechanisms could also be involved to introduce consolidation and memory transfer mechanisms. A larger and more detailed network, with multi-site plasticity (e.g. [64]) and a library of basic motor repertoires, could enhance the learning processes.

Finally, we have conformed to a general scheme, in which mossy fibers convey contextual information and climbing fibers convey instruction signals for learning. However, the real biological role of IOs is debated, ranging from carriers of error-like or of generic attention signals [65], [66]. Whatever the true biological meaning, the complex spike frequency decreases with learning resembling experimental observations [67], [68]. Recently new evidences about the mechanisms of the olivocerebellar circuit showed burst-like activities on subthreshold oscillations, going beyond the assumption of an all-or-none teaching signal, and thus linking the timing and learning aspects of motor function [69], [70]. It can be envisaged that the controller could be used to test different hypotheses on the role and functioning of IOs and climbing fibers in the future.

### Perspectives and limits of the cerebellar SNN

The neurorobot implementation had to face two crucial issues, those of implementing a real-time SNN and of connecting it with the system controller. The cerebellum SNN was built using the EDLUT simulator with exponential synapse models and leaky integrate-and-fire neurons. According to the event-driven simulation strategy proposed in [37], the behavior of each cell type with each possible condition was precompiled in look-up tables during a pre-simulation stage. The EDLUT SNN is expandable, i.e. it can be scaled and implemented with new cellular models without altering its main structure. The limitation is that the more complex are the neurons and the larger is the network, the higher are the RAM requirements. In its present configuration, the EDLUT SNN allowed cerebellum-like models with tens of thousands of cells to run in real-time in a common personal computer [71]. The transformation of the analog desired or actual states into MF spike trains was implemented by using overlapping radial basis functions [36], [72] as receptive fields of the input-variable space. These RBFs generated sparse activity patterns in the MFs that facilitated the coding in successive layers. Although this encoding strategy required a high number of fibers to encode a few variables, the use of evolutionary algorithms may be implemented in order to generate RBF settings that optimize both the information transmission and storage capacity [73].

The present cerebellar SNN was designed to emulate a single functional module, intended as the minimal unit capable of controlling a given behavior. A more complex and diversified input-output space could be emulated by considering that the cerebellum can be seen as a repetition of modules weakly interacting through mossy, climbing and parallel fiber connections [45]. Thus, in principle, this cerebellar SNN can be updated with more detailed neurophysiological properties, for example by adding new neural dynamics and learning rules, and expanded to account for multiple interconnected modules. Distributed multiple forms of plasticity observed in the cerebellar network [74], [75], [76] are required to extend the learning properties leading to robustness, acceleration, multi-scale adaptation and consolidation of memory. Distributed plasticity, nuclear and cortical, has been recently implemented in an analog cerebellar model [32] and it could be translated into the real-time EDLUT simulator by applying appropriate and optimized information-coding strategies, thus enriching the cerebellar SNN with multi-site plasticity.

This update may allow us to achieve a higher level of realism, extending the neurorobot functionalities over a rich and redundant multi-sensory space and a multi-joint output space [77], [78]. A large-scale network of the cerebellum representing multiple modules in detail would require high performance computing, hardware accelerators and neuromorphic circuits [79], [29], [27], [80] in order to be compatible with the compact design and real-time requirements of neurorobotic applications.

## Conclusions

We have efficiently linked low-level brain circuits with high-level functions integrating a SNN into a neurorobot operating in real-time. The main added value of learning in our neurorobot is that it succeeded in reproducing how biological systems acquire, extinguish and express knowledge of a noisy and changing world, in multiple cerebellum-driven learning tasks performed by a real robot moving in perturbed environments. The real world is always more noisy than the worst case simulation can accomplish [81], and learning is actually a long-lasting experience-dependent change in behavior, which can realistically be observed only from an embodied system.

It is envisaged that improving the realism of the SNN will allow us to make predictions about the nature of implicit computations occurring in the cerebellar SNN. This approach has the challenging potential to represent the initial building block of a more complex brain-inspired controller, into which other realistic SNNs emulating different brain structures involved in sensorimotor integration could be embedded.

As broader robotic implications, semi-autonomous robots can take advantage of cerebellar-inspired building blocks in their control system whether it allows them to adapt to multiple context-dependent behaviors [12].
