# Correction: Adaptive Robotic Control Driven by a Versatile Spiking Cerebellar Network

**DOI:** 10.1371/journal.pone.0118518

**Published:** 2015-03-05

**Authors:** The PLOS ONE Staff

The URL that links to the computational models is incorrect in both the Data Availability statement and in the Methods section. The correct link to access the data is: http://senselab.med.yale.edu/modeldb/ShowModel.asp?model=167414.
